# From evidence to action: identification and strategic application of cholera priority areas for multisectoral interventions (PAMIs) in Sudan (2016–2025)

**DOI:** 10.3389/fpubh.2026.1728112

**Published:** 2026-03-05

**Authors:** Dalya Eltayeb, Ahmad Izzoddeen, Mazza Abasher, Asia Dahab, Adam Eissa, Catherine Makwe, Elfadil Mohammed Mahmoud

**Affiliations:** 1 Directorate General of Primary Health Care, Federal Ministry of Health, Khartoum, Sudan; 2 Directorate General of Health Emergencies and Epidemic Control, Federal Ministry of Health, Khartoum, Sudan; 3 International Federation of Red Cross and Red Crescent Societies, Addis Ababa, Ethiopia

**Keywords:** cholera, complex humanitarian emergency, conflict, GTFCC, National Cholera Plan, oral cholera vaccine (OCV), priority areas for multi-sectoral interventions (PAMIs)

## Abstract

**Background:**

Sudan has a long history of struggling with recurrent cholera outbreaks. The interplay of infrastructure damage, massive displacement, and limited access to safe water and sanitation has increased the risk of cholera transmission. The study aims to identify priority areas for multisectoral interventions (PAMIs) to inform public health policy and ensure evidence-based strategic control of cholera in Sudan.

**Methods:**

In this retrospective study, national cholera data from 2016 to 2024 (excluding 2020–2022) were used. The Global Task Force on Cholera Control (GTFCC) tool for identifying PAMIs was applied to the analysis. Key epidemiological indicators, such as incidence, mortality, and persistence of cholera, were used to score each geographical unit and were subsequently summed to generate a priority index (PI). Furthermore, vulnerability factors were evaluated, including water, sanitation, and hygiene (WASH) infrastructure, crowded settings, displacement, inaccessibility, and community awareness. A national stakeholder validation workshop reviewed and contextualized the findings to finalize the list of PAMIs. This study also documents the immediate applications of PAMIs for strategic cholera control.

**Results:**

A priority index threshold of 6 was established through stakeholders’ consensus, which resulted in the identification of 52 PAMIs. These administrative units account for 80% of reported cholera cases and 77% of deaths, encompassing 31.5% of Sudan’s total population. The identified PAMIs were immediately applied to guide preventive oral cholera vaccine (OCV) planning, resulting in the selection of 40 high-risk localities and the development of Sudan’s first national 3-year OCV plan. In addition, they informed the development of the updated National Cholera Plan (NCP; 2025–2030) as a long-term strategy for cholera control.

**Conclusion:**

The study provides strong evidence-based identification of PAMIs in Sudan and supports local, national, regional, and global efforts to control cholera risk. Targeted interventions in these areas are essential to contain outbreaks and build resilient public health systems. The PAMIs identification exercise informed evidence-based prioritization and planning for preventive OCV deployment and catalyzed the establishment of a national committee that developed the updated national cholera plan (2025–2030), reflecting the country’s shift toward proactive and sustainable cholera prevention. Sudan’s experience demonstrates the feasibility and policy relevance of the PAMIs framework, even in highly fragile humanitarian settings.

## Introduction

Cholera remains a major public health challenge in Sudan, characterized by recurrent outbreaks that are increasingly difficult to control due to armed conflict, environmental hazards, and systemic infrastructure collapse. The current civil conflict, which erupted in April 2023, has severely undermined health system functionality and worsened health vulnerabilities across the country. This has caused one of the world’s largest displacement crises, affecting millions of people who have been pushed to live in poor conditions with limited access to safe water, sanitation, and hygiene (WASH) (1, 2). Healthcare services have been significantly disrupted, with a considerable number of facilities either destroyed or operating at reduced capacity due to insecurity, staff shortages, and supply chain disruptions. The situation is further worsened by seasonal flooding, which either exacerbates the contamination of existing drinking water sources or pushes people to use alternative, unsafe sources (3). Despite the recent widespread cholera outbreaks (3, 4), the Ministry of Health (MoH) and partners have made significant efforts in responding to cholera, including oral cholera vaccine (OCV) campaigns in high-risk zones (5, 6). However, a more strategic, evidence-based approach is needed for long-term control and, ultimately, the elimination of cholera risk, particularly in fragile settings such as Sudan.

One of the important steps in this direction was the cholera hotspot mapping exercise conducted in 2021, which identified 12 priority localities across six states using epidemiological data. However, this analysis is currently outdated and does not provide a sufficient basis for effective planning, given recent changes in transmission dynamics and emerging vulnerabilities.

For a more updated analysis that incorporates recent changes, Sudan undertook its first-ever national identification of priority areas for multisectoral interventions (PAMIs). This exercise was conducted amid the ongoing complex humanitarian emergency, aiming to update the cholera risk profile based on the latest available surveillance and contextual vulnerability data. The findings provide critical evidence that guides the development of the National Cholera Plan (NCP), strategically informs multisectoral resource allocation, and hopefully contributes to achieving the Global Roadmap goal to end cholera by 2030 (7). This exercise is critical for informing and guiding the country’s efforts to control the persistent public health risks posed by cholera. By using the most recent Global Task Force on Cholera Control (GTFCC) methodology, this study ensures data reliability and aligns with global standards for cholera mapping. Furthermore, previous hotspot mapping had become outdated and lacked precision, as it relied solely on two epidemiological indicators and failed to incorporate essential vulnerability factors in the changing cholera landscape in Sudan.

This article documents the PAMI identification exercise in Sudan, highlighting its methodology and presenting the results. It provides valuable insights into how evidence-based targeting can be implemented even in highly fragile, conflict-affected settings. The endorsed PAMIs constitute a critical tool for strengthening and advancing cholera prevention, preparedness, and response in Sudan and other similar contexts, both regionally and globally.

## Methods

The PAMI identification exercise followed a step-by-step analytical workflow. The analysis began with the preparation of administrative level 2 (locality) cholera surveillance data. Subsequently, epidemiological indicators were calculated for each locality and scored using the 2023 GTFCC PAMI method for the cholera control. The third step involved generating a priority index (PI) by summing the individual indicator scores, which was followed by an assessment of vulnerability factors to provide the necessary contextualization. Finally, the results were reviewed and validated during a national workshop by a multisectoral coordination team comprising national and subnational stakeholders.

### Study design and settings

This study documents the PAMI identification exercise conducted in Sudan by the Federal Ministry of Health (FMoH). The country utilized the GTFCC methodology through a retrospective analysis of cholera data from the national surveillance database covering the period 2017–2024, complemented by a review of vulnerability factors. The analysis was structured into two main steps: (1) quantitative scoring using the GTFCC Excel-based PAMI tool, and (2) validation by national and subnational stakeholders.

### Data sources and geographic units

Administrative level-2 units, corresponding to localities within the governance structure in Sudan, were used as the geographic units of analysis. These units are operationally consistent with the National Cholera Plan (NCP) implementation and provide an optimal scale for multisectoral interventions.

Population estimates were based on projections by the United Nations Office for the Coordination of Humanitarian Affairs (OCHA) for 2022 and 2024, which were adjusted at the locality level using national demographic growth rates and further adjusted to account for displacement movement.

Epidemiological data were drawn from Sudan’s national surveillance cholera database. The analysis covered the period from 2016 to 2024. The analysis excluded the years 2020–2022, as recommended by the GTFCC, because no cholera was reported during that period.

### Cholera case definition and reporting

Sudan’s surveillance system defines a suspected cholera case as *“any patient aged 5 or older presenting with a sudden onset of watery diarrhea, without abdominal pain, with or without vomiting.”* The presence of severe dehydration or death from acute watery diarrhea classifies the case as probable. Confirmation requires the identification of *Vibrio cholerae* through culture or RT-PCR. Rapid diagnostic tests are used to support detection, with confirmatory testing performed on a few cases at the beginning of outbreaks.

Cases were reported from health facilities and communities to the locality (district) level, where rapid response teams (RRTs) conducted initial investigations, including case documentation, contact tracing, and environmental and stool sample collection (4). Reports were subsequently sent to the state level for validation and further support and then to the national level for final validation and compilation. Stool samples were tested at the National Public Health Laboratory (NPHL), with feedback sent to the lower levels. Environmental (drinking water) samples were collected by RRTs and tested at the state-level environmental health directorates using total coliform and *E. coli* tests.

### PAMI scoring methodology

According to the GTFCC guidelines, the following indicators were used to measure the priority index (PI): incidence rate (per 100,000 population), mortality rate, and persistence of cholera (weeks with reported cases). Each indicator was scored on a scale of 0–3, based on the statistical distribution (median and 80th percentile thresholds).

Then, the PI was calculated by summing these three scores:


PI=Incidence Score+Mortality Score+Persistence Score

Note: The testing coverage indicator was assessed but excluded from the final index due to low representativeness. According to the GTFCC guidelines, it was considered insufficient because fewer than 80% of geographical units had testing coverage in at least 50% of reporting weeks.

### Vulnerability assessment

A total of seven vulnerability factors were considered: humanitarian emergency classification, access to WASH services, population at risk, population movements, insecurity, accessibility, and expert insights. The data sources for the assessment included Sudan’s 2014 Multiple Indicator Cluster Survey (MICS), the 2018 S3M national survey, and information provided by field experts during the stakeholder workshop. In addition, the vulnerability factor assessment was conducted through secondary data analysis. This involved a review of the 2025 Situational Analysis Report from the Directorate of Environmental Health, supplemented by findings from the 2023 WHO/UNICEF Joint Monitoring Programme (JMP). The data were extracted from the respective reports and entered into the GTFCC tool; notably, no primary data were generated for this exercise.

### Stakeholder validation workshop

A hybrid (both virtual and in-person) validation workshop was conducted for 3 days (28 to 30 April 2025), attended by more than 120 participants (90 in person and 30 virtual) from 11 states. The workshop was organized by the FMoH with support from the International Federation of Red Cross and Red Crescent Societies (IFRC), WHO, UNICEF, and national partners. The workshop goal was to validate the PAMI findings, resolve discrepancies, and finalize the country’s list of priority areas. Participants were engaged in plenary sessions and small group discussions. Participants were grouped based on geographical origin, role, and technical area. The stakeholders came from different sectors, including government (federal and state Ministries of Health, Ministry of Irrigation and Water Resources), health system actors (technical staff from surveillance, emergency response, immunization, laboratory, risk communication, WASH, and curative medicine departments), and key humanitarian actors (the WHO, IFRC, UNICEF, and national and international NGOs). This diversification ensured the multisectoral nature of the validation, extending beyond health alone. Decision-making was based on consensus, with participants agreeing on the priority index threshold (PI ≥ 6) through structured discussion and relying heavily on contextual knowledge to interpret the results.

### Map creation

The maps were created using the QGIS software (version 3.x) with the geodetic datum and coordinate system WGS 84 (EPSG:4326). We found the selected resolution to be appropriate for national-level planning because we used administrative level 2 (locality-level) polygon shapefiles from humanitarian data sources. Polygon-based choropleth mapping was used, with incidence and priority indices used to assign values to each locality polygon.

### Immediate application review

In addition, this study documents the immediate application of the identified PAMIs in shaping national cholera control actions. Particular attention was given to how the PAMI outputs informed preventive OCV planning and the development of the National Cholera Plan (2025–2030).

## Results

### Trends and burden of cholera in outbreak-affected areas

**Figure 1:**
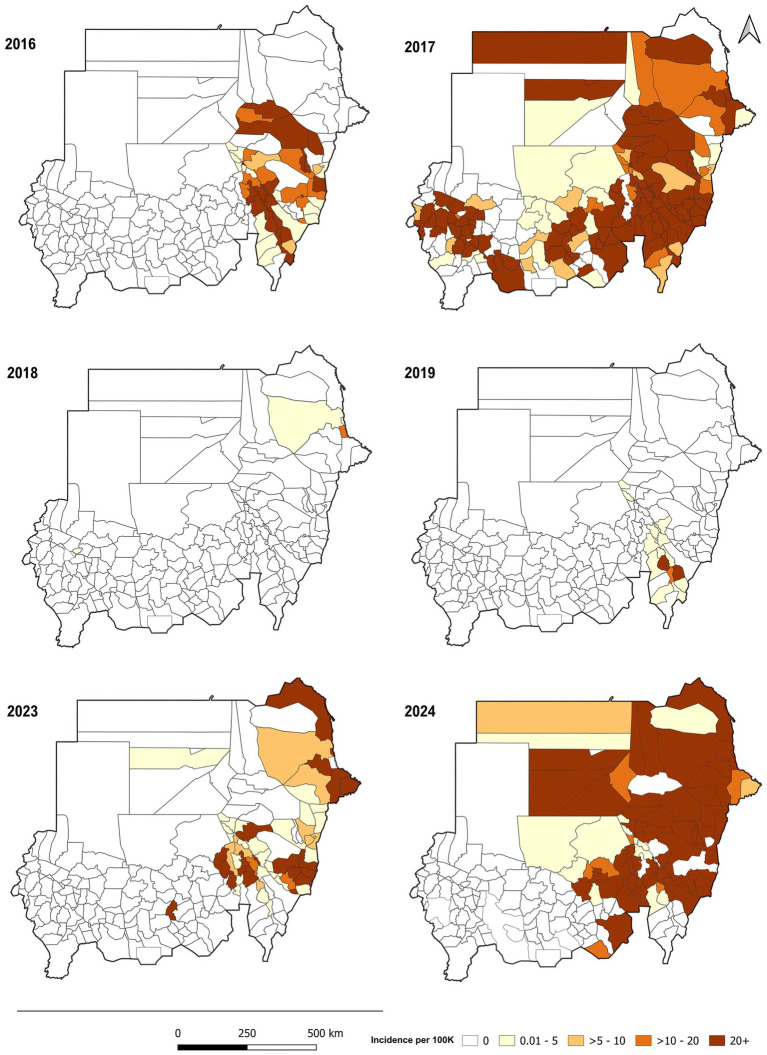
Distribution of cholera cases by locality for each analysis year, Sudan, 2016–2024.

**Figure 2:**
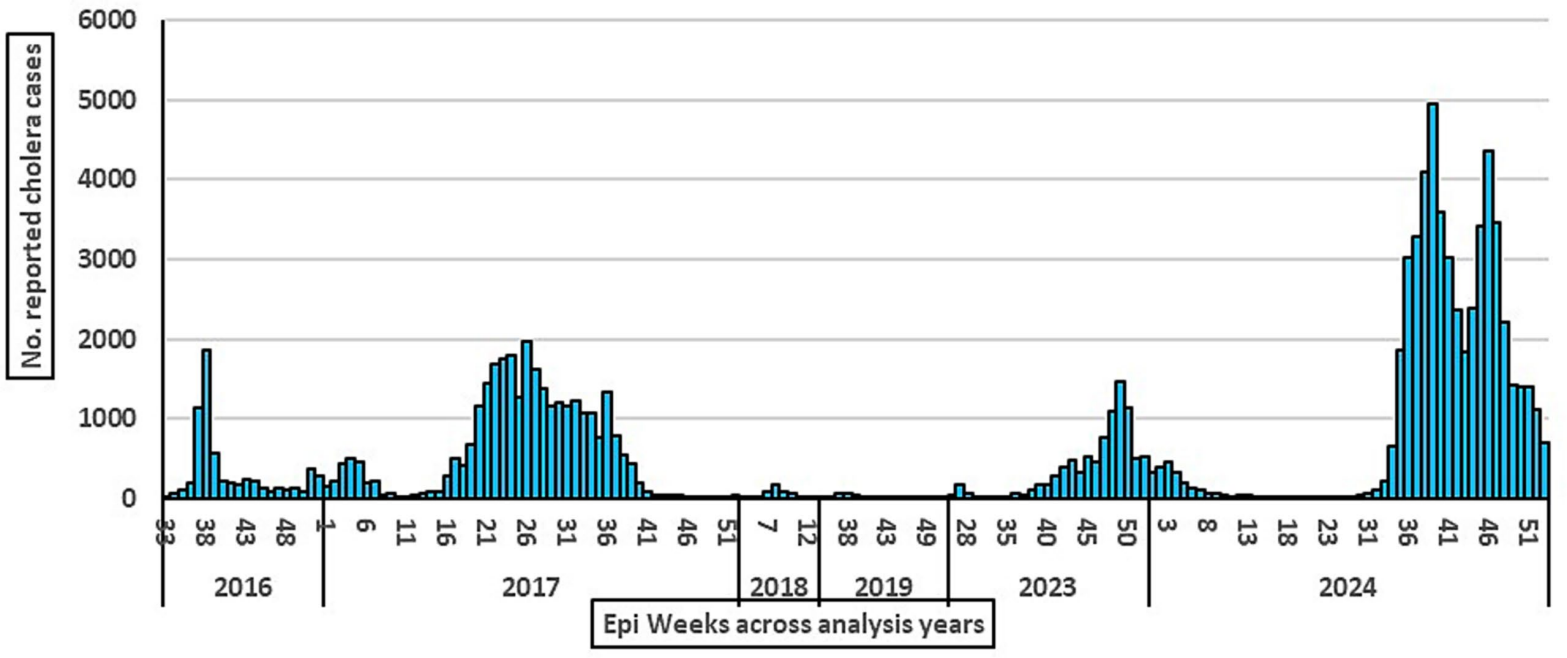
Epidemic curve of cholera across the analysis period, Sudan, 2016–2024.

During the analysis period, cholera showed remarkable persistence and recurrence in the target years (Figures 1, 2), causing significant morbidity and mortality across a wide geographical spread and demonstrating a clear variation (Figure 2). The total number of localities with at least one reported cholera case accounted for (% of total localities) across the states. Based on GTFCC tool measurements, the median incidence was 27.2 cases per 100,000 person-years, the median mortality was 1.08 per 100,000 person-years, and cholera persistence was observed during 5.8% of weeks with at least one reported case.

### Testing representativeness

During the analysis period, only 17 NCP operational geographical units (representing 11.3% of units with at least one reported cholera case) achieved a weekly testing coverage of ≥ 50%. In contrast, 93 units (representing 61.6%) demonstrated some level of testing coverage (> 0%). These findings indicate an insufficient level of representativeness of cholera testing; consequently, this indicator was excluded from the final analysis.

### Priority index and geographic coverage

The prioritization exercise concluded with the selection of 52 localities across 14 states, all of which were designated as PAMIs based on a priority index threshold of ≥6. The identified PAMIs represent approximately 31.5% of Sudan’s population and accounted for 80.2% of all reported cholera cases and 77.4% of cholera-related deaths during the analysis period. Full details on the distribution by priority level, population, and disease burden are presented in Table 1.

**Table 1:** Summary of endorsed PAMIs by priority index, population, case load, and cholera-related death, Sudan, (2016–2024).

Priority index	# localities	# states	Population (% of total)	Cumulative cases (% of total)	Cumulative deaths (% of total)
9	8	4	1,500,373 (3.5%)	114,119 (14.2%)	324 (12.8%)
8	16	9	5,680,656 (13.2%)	41,897 (42.1%)	940 (37%)
7	14	9	2,919,414 (6.8%)	12,974 (13%)	390 (15.4%)
6	14	10	3,487,159 (8.1%)	10,805 (10.9%)	311 (12.3%)
Cumulative	52	14	3,487,159 (31.5%)	79,795 (80.2%)	1,965 (77.4%)

**Figure 3:**
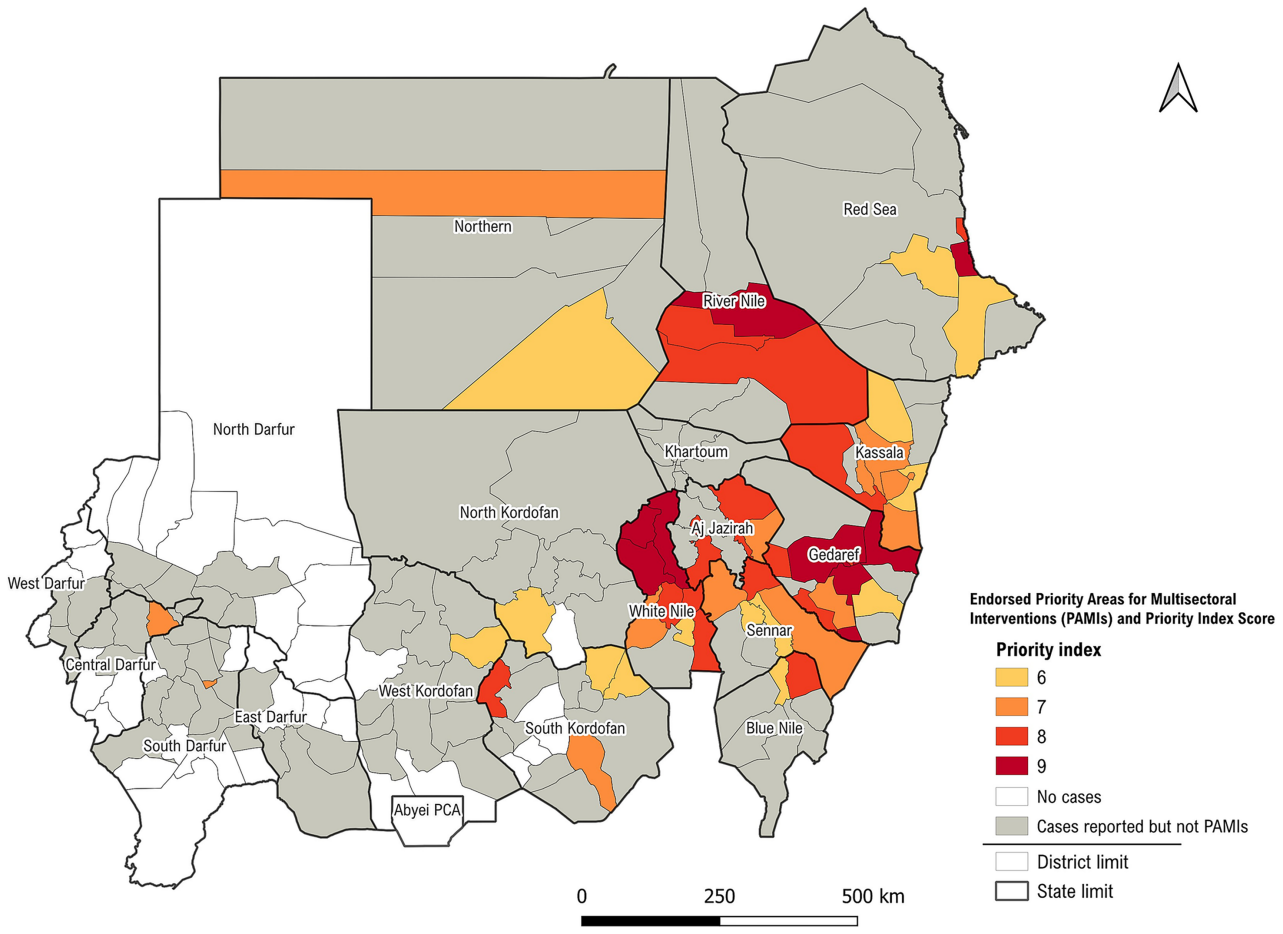
Endorsed priority areas for multisectoral interventions (PAMIs), Sudan, 2025.

Before reaching a consensus on the 52 PAMIs, the committee and stakeholders discussed the inclusion of five additional localities with a priority index below six, based on contextual knowledge and the presence of vulnerability factors. This inclusion increased the total number of PAMIs to 57 (representing 37% of the population, 91% of cholera cases, and 90% of deaths). However, the committee ended up making a decision to retain the initial list without adding additional PAMIs to remain consistent with the GTFCC recommendation, which advise that selected PAMIs should cover up to 30% of the total population (see Figure 3).

### Immediate applications of PAMIs

#### Application of PAMIs in preventive OCV planning

PAMIs provided a critical foundation for Sudan’s preventive OCV planning. The FMoH used the GTFCC prioritization tool as a structured approach to identify eligible areas for OCV deployment within the approved PAMIs. Localities were classified based on two main indicators: (1) reported cholera incidence in at least 40% of the PAMI analysis years and (2) a mean annual incidence of ≥10 cases per 100,000 population. Additional criteria were applied to borderline localities, including the frequency of outbreaks over the past 5 years. The outcome was the selection of 40 of the 52 PAMIs as eligible for preventative vaccination, targeting a population of 14.8 million. This, in turn, led to the development of the national 3-year OCV plan and the preparation and submission of preventative OCV requests for the first time in Sudan. By grounding vaccine prioritization in epidemiological evidence, the use of PAMIs has optimized both the efficiency and equity of cholera prevention efforts in Sudan.

#### Institutional application: development of the updated National Cholera Plan (2025–2030)

The identification of PAMIs directly initiated and guided action for Sudan’s long-term cholera control strategy, referred to as the National Cholera Plan (NCP). This institutional exercise spanned approximately 6 months and resulted in an evidence-based, comprehensive policy document. Immediately following the validation of the PAMIs by the GTFCC, the FMoH established a national committee to develop the updated National Cholera Plan (NCP) for 2025–2030. The committee was composed of representatives from the FMoH (all relevant departments), WHO, IFRC, UNICEF, and other national and international partners. The committee used the PAMI results as the primary evidence base for defining geographical and contextual priorities and adopted them as the geographical framework for developing the National Cholera Plan.

The PAMIs provided a clear framework for identifying PAMIs, enabling alignment of surveillance, health system strengthening, WASH, and vaccination components within a single, evidence-based strategy. This institutional process represented a clear shift from reactive responses to a proactive and sustainable approach tocholera prevention and control. The endorsed PAMIs enabled the prioritization of limited resources toward areas with the highest cholera burden to achieve the highest impact in reducing mortality and morbidity. This prioritization will guide the efficient allocation of preventive OCV, surveillance strengthening, and WASH interventions, while reducing fragmentation and duplication of efforts.

## Discussion

Cholera is a top public health priority in Sudan, causing significant morbidity and mortality between 2016 and 2024. The specific patterns and clustering of cases within the identified PAMIs indicate persistent rather than sporadic transmission. Furthermore, this persistence reflects cholera endemicity in Sudan, as opposed than isolated outbreaks. These findings underscore the urgent need for targeted, geographically focused intervention planning. Similar prioritization exercises conducted in Kenya and Cameroon, utilizing the updated GTFCC guidelines, demonstrated comparable patterns of clustering, where the cholera burden was concentrated in a limited number of administrative units. These results underscore the importance of directing limited resources toward high-risk areas to maximize efficiency and public health impact (8, 9).

The risk has further increased in recent times due to the ongoing complex emergency, which has increased vulnerabilities and contributed to a higher incidence and case fatalities (3, 5). During the analysis period, an apparent absence of cholera was observed, coinciding with the peak of the COVID-19 pandemic. This likely does not reflect a true absence of the disease, but may instead be attributed to weakened surveillance and response mechanisms, as resources were heavily diverted to managing COVID-19 (10). These factors indicate that cholera requires a more strategic multisectoral approach to achieve effective control and, hopefully, elimination in the near future.

In Kenya and Cameroon, similar exercises were conducted in relatively stable contexts, with broader laboratory coverage and the inclusion of testing indicators (8, 9). In contrast, the Sudan identification exercise was conducted amid a complex humanitarian emergency characterized by conflict, displacement, fragmented surveillance, and limited laboratory capacity. The uniqueness of the Sudanese experience lies in conducting this exercise amid active conflict, significant access constraints, and limited testing representativeness. Despite these persistent challenges, the country successfully identified a comparable geographical mapping of the disease burden in specific localities, demonstrating the feasibility and policy relevance of the PAMI framework even in highly fragile and insecure settings.

The immediate use of the results to inform policy and planning demonstrates agility in leveraging data to directly guide time-bound preventive OCV campaigns and the development of a national cholera plan. This provides a practical model for other cholera-affected countries operating in fragile and conflict-affected contexts. The identification of PAMIs using the GTFCC methodology offers a strategic approach for targeting cholera interventions in Sudan. This critical exercise is important, particularly in resource-constrained settings, to implement targeted interventions in the highest-risk areas (7). The identified PAMIs represent approximately 31.5% of Sudan’s population and accounted for 80.2% of all reported cholera cases and 77.4% of cholera-related deaths, representing the highest cholera burden and vulnerability. This stratified distribution underscores the strong association between the priority index and cholera burden, providing a rationale for the targeted allocation of multisectoral interventions in these high-risk localities.

Implementing targeted multisectoral interventions in these areas is expected to result in a significant reduction in cholera mortality and, ideally, contribute positively toward achieving the Global Roadmap goals (11). The results underscore the importance of basing resource allocation on a combination of epidemiological evidence and contextual vulnerability assessments. Localities such as Wad Elhilaw in Kassala and Galabat in Gadaref (scored 9) are strongly associated with persistent cholera transmission and are recognized as frequent sources of outbreaks (4, 5). Strategic investments in areas such as these are a top priority and critical to effective long-term control.

Although the surveillance database was highly valuable in finalizing the PAMI list, further efforts are needed to strengthen testing capacity and improve analytical accuracy. The level of cholera testing representativeness in Sudan during the analysis period was considered insufficient, as it fell below the GTFCC threshold, which requires that at least 80% of affected geographical units have ≥ 50% weekly testing coverage during the period of reported cholera cases.

Embedding PAMIs within the national cholera control framework improved resource allocation efficiency and facilitated a shift from reactive responses to a more strategic and coordinated cholera prevention approach. Addressing cholera remains a collective responsibility, rather than the burden of a single entity. This requires the coordinated efforts of all actors, from national leadership to sub-national stakeholders, to ensure effective outbreak control and the implementation of sustainable, long-term prevention measures (12).

### Limitations

The limitations of this study were primarily driven by the conflict-affected context. These limitations include significant data gaps, particularly during the conflict years (2023–2024), resulting from geographical inaccessibility and disrupted reporting mechanisms. In addition, limited laboratory capacity led to suboptimal testing representativeness, which necessitated the exclusion of the testing indicator from the priority index. Furthermore, reliance on projected population estimates may have affected the precision of incidence calculations. Finally, the contextual constraints, including pervasive insecurity and the collapse of communication networks in several states, hindered the participation of a sufficient number of stakeholders from these regions during the validation process.

## Conclusion

This study provides critical evidence for informed decision-making and enables more strategic planning and implementation of cholera control activities. The use of the GTFCC PAMI methodology ensured a rigorous, standardized approach to risk prioritization. The observed cholera patterns indicate persistent structural vulnerabilities and regional endemicity within Sudan. The identified PAMIs comprised 52 localities across 14 states, representing a wide geographical coverage of areas with the highest cholera burden. The integration of epidemiological analysis with stakeholder validation fostered national consensus and strengthened ownership of the results. The PAMI identification exercise not only informed evidence-based prioritization for preventive OCV deployment but also led to the establishment of a national committee to develop the updated NCP (2025–2030), marking a decisive shift toward proactive and sustainable cholera prevention. Ultimately, the Sudan experience demonstrates the feasibility and policy relevance of the PAMI framework, even in highly fragile and insecure settings.

## Data Availability

The data analyzed in this study is subject to the following licenses/restrictions: data is in the archive of Sudan Federal Ministry of Health and only available by the first author upon request. Requests to access these datasets should be directed to dalyaeltayeb@gmail.com.
